# Adjuvant nivolumab after chemoradiotherapy and resection for patients with esophageal cancer: A real‐world matched comparison of overall survival

**DOI:** 10.1002/ijc.70168

**Published:** 2025-09-23

**Authors:** Rob H. A. Verhoeven, Steven C. Kuijper, Marije Slingerland, Bas Wijnhoven, Mark I. van Berge Henegouwen, Peter S. N. van Rossum, Sarah Derks, Bianca Mostert, Nadia Haj Mohammad, Hanneke W. M. van Laarhoven

**Affiliations:** ^1^ Department of Medical Oncology Amsterdam UMC Location University of Amsterdam, Medical Oncology Amsterdam The Netherlands; ^2^ Cancer Center Amsterdam Cancer Treatment and Quality of Life Amsterdam The Netherlands; ^3^ Department of Research & Development Netherlands Comprehensive Cancer Organisation (IKNL) Utrecht The Netherlands; ^4^ Department of Medical Oncology Leiden University Medical Center Leiden The Netherlands; ^5^ Department of Surgery Erasmus Medical Center Rotterdam The Netherlands; ^6^ Department of Surgery Amsterdam UMC Location University of Amsterdam, Medical Oncology Amsterdam The Netherlands; ^7^ Department of Radiation Oncology Amsterdam UMC Amsterdam The Netherlands; ^8^ Oncode Institute Amsterdam The Netherlands; ^9^ Department of Medical Oncology Erasmus MC Cancer Institute Rotterdam The Netherlands; ^10^ Division of Imaging and Cancer Utrecht University Medical Center, Utrecht University Utrecht The Netherlands

**Keywords:** adjuvant therapy, esophageal cancer, nivolumab, propensity score matching, real world data

## Abstract

The Checkmate‐577 trial showed a significant disease‐free and a non‐significant overall survival benefit for nivolumab compared to placebo in esophageal or gastroesophageal junction (GEJ) cancer patients with residual disease after neoadjuvant chemoradiotherapy (nCRT) and resection. Real‐world overall survival (OS) data has not yet been presented. The aim of this study was to evaluate OS of patients treated with or without adjuvant nivolumab in a nationwide real‐world matched comparison.For this study, patients diagnosed with non‐metastatic esophageal or GEJ cancer in 2020–2023 who had residual pathological disease after nCRT and resection were selected from the Netherlands Cancer Registry. 333 patients received treatment with adjuvant nivolumab. From the period before the introduction of nivolumab, 486 patients were selected who received nCRT and resection alone. Propensity score trimming and nearest neighbor matching were used to create two well‐balanced groups of 311 patients per treatment group. Median follow‐up time was 24.4 months and 31.4 months for patients treated with and without adjuvant nivolumab, respectively. The 2‐year OS was 66.8% (95% confidence interval [CI]: 61.6%–72.44%) and 58.8% (95% CI: 53.5%–64.5%) for the groups with and without nivolumab, respectively (log‐rank *p* = 0.024), hazard ratio: 0.75, 95% CI: 0.60–0.97 (*p* = 0.024). In conclusion, this matched real‐world study showed an OS in favor of patients treated with nivolumab compared to patients without nivolumab. This represents the first report on a real‐world OS benefit in this setting. As follow‐up and the number of events are still limited, these analyses should be interpreted with caution and updated in the forthcoming years.

## What's New?

Patients with esophageal or gastroesophageal junction (GEJ) cancer with residual disease after treatment with neoadjuvant chemoradiotherapy (nCRT) and resection frequently experience poor survival. Improvements in outcome, however, may be possible using adjuvant treatment with nivolumab. In this study, the authors gathered real‐world data on overall survival for patients treated with adjuvant nivolumab to better understand survival outcomes reported from clinical studies. Overall survival was significantly higher for patients treated with nivolumab than for those without. The findings highlight the potential of adjuvant nivolumab and call for further investigation of its toxicity and cost trade‐offs in real‐world settings.

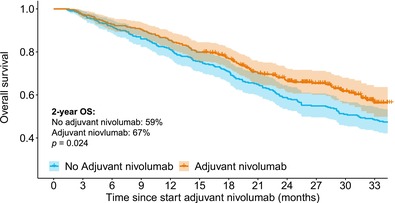

**Abbreviations**
BMI: Body mass index
CI: Confidence interval
DFS: Disease‐free survival
DMFS: Distant metastasis‐free survival
GEJ: Gastroesophageal junction
IQR: Interquartile range
NCR: Netherlands Cancer Registry
nCRT: Neoadjuvant chemoradiotherapy
OS: Overall survival
SMD: Standardized mean difference

## 1 INTRODUCTION

Annually, more than 600,000 people are diagnosed with esophageal cancer worldwide, which is projected to increase to over 950,000 diagnoses in 2040.^1^ Despite therapeutic improvements in the past decade, survival of patients with esophageal cancer remains low.^1^, ^2^ After publication of the CROSS trial, neoadjuvant chemoradiotherapy (nCRT) followed by surgical resection has become a standard of care for patients with locally advanced resectable esophageal or gastroesophageal junction (GEJ) cancer in most Western countries.^3^, ^4^ However, survival of patients with residual disease after treatment with nCRT is poor, underscoring the potential benefit of adjuvant treatment for these patients.^5^, ^6^

The randomized controlled CheckMate 577 trial investigated whether adjuvant immunotherapy with nivolumab for patients with esophageal or gastroesophageal junction (GEJ) cancer with pathologically confirmed residual disease after previous treatment with nCRT and resection would increase survival.^7^ The trial showed a significantly longer median disease‐free survival (DFS) of 22.4 months in the group who received adjuvant treatment with nivolumab compared to a median DFS of 11.0 months in patients who were treated with placebo (*p* < 0.001).^7^ Median distant metastasis‐free survival (DMFS) was 28.3 and 17.6 months in the nivolumab and placebo groups, respectively.^7^ In May 2025, the results on overall survival (OS), a secondary outcome measure of CheckMate 577, were presented at the annual ASCO conference. Median OS was numerically longer in the adjuvant nivolumab arm (51.7 months) compared to the placebo arm (35.3 months), with a 5‐year OS of 46% in the adjuvant nivolumab group and 40% in the placebo group, but this did not reach statistical significance.^8^ In patients with CPS ≥1 in the resection specimen, which comprised 88% of the study population, OS was 45.5 months in the nivolumab group and 33.5 months in the placebo group, with a significant HR of 0.79.^8^

The combination of a significant DFS benefit and a non‐significant but considerable OS benefit for the population as a whole complicates the interpretation of the outcomes of CheckMate 577 for daily patient care. Taking into account that findings from clinical trials may not consistently yield similar benefits in daily clinical practice.^9^, ^10^ It remains unclear whether adjuvant treatment with nivolumab will result in a clinically relevant OS benefit for patients with esophageal or GEJ cancer in routine clinical care, while nivolumab treatment can be associated with sometimes significant toxicity and costs.

Therefore, the aim of the current study was to conduct an initial assessment of OS among patients who received adjuvant nivolumab after nCRT and resection compared to patients who underwent nCRT and resection alone in a comprehensive nationwide real‐world matched comparison.

## 2 METHODS

### 2.1 Patient population

The Netherlands Cancer Registry (NCR) is a nationwide population‐based database in which all Dutch inhabitants with a diagnosed malignant cancer are registered. On November 23, 2023, we extracted data of completely registered patients with non‐metastatic esophageal, GEJ, or cardia cancer diagnosed between 2020 and 2023 from the NCR. At that time, all Dutch patients with diagnosed esophageal, GEJ, or cardia cancer in 2020 and 2021, and 72% and 3% of the patients diagnosed in 2022 and 2023, respectively, were completely registered in the NCR. Patients had to be treated with neoadjuvant chemoradiotherapy (nCRT) consisting of carboplatin and paclitaxel^11^ and a surgical resection. In order to select a group of patients that would have been eligible for adjuvant nivolumab treatment, patients with complete pathological response (ypT0N0), distant metastases within 14 weeks after resection, or patients deceased within 14 weeks after resection were excluded. As the EMA indication, in contrast to the CheckMate 577 trial, does not exclude patients with irradical resection (R1/R2), we have also not excluded this group from the current study.

Nivolumab was initially reimbursed for adjuvant treatment in esophageal or GEJ cancer patients in the Netherlands starting from January 1, 2022. However, reimbursement ceased as of January 1, 2024, following adjustments in Dutch guidelines related to reimbursement based on DFS outcomes. Therefore, all patients from the previously described group who started with adjuvant nivolumab treatment since January 1, 2022, were classified into the nivolumab group. To select a group of patients that would have been eligible for adjuvant treatment with nivolumab had it been available at the time, we selected all patients fulfilling the previously described criteria and who were treated with nCRT and a surgical resection prior to the November 1, 2021, but were not treated with nivolumab. Patients who underwent a surgical resection on or later than the November 1, 2021, were considered potential candidates for treatment with nivolumab, given that adjuvant nivolumab treatment typically commences more than 2 months after resection.

Information on vital status was obtained through annual linkage with the Dutch Personal Records Database and updated until January 31, 2025.

### 2.2 Statistical analyses

Propensity score matching was used to create two comparable and balanced groups of patients. Three medical oncologists (MS, NHM, and HL) and two scientific researchers (RV and SK) selected a set of 18 patient (age, sex, Body Mass Index [BMI], Charlson comorbidity index, performance status prior to nCRT, and other malignancies), tumor (primary tumor location, histology, differentiation grade, ypT, and ypN), and treatment‐related variables (number of chemotherapy cycles, radiotherapy dose, time between nCRT and resection, type of surgery, annual resection volume of hospital, surgical radicality, and post‐operative complications) based on expert knowledge about factors that might influence the choice for whether or not to start adjuvant treatment with nivolumab, but also factors that might influence OS. These variables were used to estimate the propensity of receiving adjuvant nivolumab using a multivariable logistic regression model. Propensity score trimming was used to remove any non‐overlapping propensity scores. After trimming, we iteratively tested five different matching methods (nearest neighbor, optimal matching, full matching, generic matching, and coarsened exact matching) and calipers (ranging from 0.01 to 0.20 times the standard deviation of the logit of the propensity score) without replacement, to evaluate which method resulted in the largest number of matched patients while maintaining a maximum allowable univariable covariate balance. We defined a standardized mean difference (SMD) of ≤0.1 as an appropriate univariable covariate balance.

To make the overall survival (OS) analyses as comparable as possible to the survival analyses of the CheckMate 577 trial, OS was calculated since the start of adjuvant treatment. For patients who did not receive adjuvant treatment, this start date was obviously nonexistent. To ensure similar starting points to make a valid OS comparison, we based the starting point for patients not treated with adjuvant therapy on the number of days between resection and adjuvant treatment of the patient to whom they were matched in the propensity score matching. Kaplan–Meier curves and the univariable hazard ratio from Cox regression analysis were tested for significance using two‐sided tests with an alpha level of 0.05. Subgroup analyses with univariable hazard ratios were conducted for histology, ypN status, and surgical radicality, as the distribution of patients across histology and ypN status was considerably different in the current study compared to the CheckMate 577 study, and as patients with R1/R2 surgical radicality may be treated in daily clinical practice but were not included in the CheckMate 577 study. To investigate the potential differential effect for adjuvant nivolumab within these subgroups, we estimated and tested the hazard ratio of the interaction effect. Missing data were imputed with a random forest imputation using the missRanger package, and the propensity score matching was performed using the MatchIt package.^12^, ^13^ A sensitivity analysis was performed to assess the robustness of the treatment effect for unobserved confounding, for which the *E*‐value was used.^14^ The *E*‐value reflects the magnitude that an unobserved confounder would need to have to negate the estimated treatment effect. The *E*‐value in this analysis can be interpreted similarly to a hazard ratio. All analyses were performed by Steven Kuijper in R version 4.3.

## 3 RESULTS

Prior to matching, 333 patients were identified who received adjuvant nivolumab after nCRT and resection and 485 patients were identified who received nCRT and resection alone (Table 1). Propensity score trimming resulted in the exclusion of 10 patients (1 with and 9 without nivolumab). Nearest neighbor matching with a caliper of 0.19 resulted in the largest number of matched patients (308 patients in each arm) while all univariable SMDs were ≤0.1 (Table 2). All patient, tumor, and treatment‐related variables were well balanced in the matched groups.

**TABLE 1:** Patient characteristics of both treatment arms prior to imputation and propensity score matching.

	No Adjuvant nivolumab	Adjuvant nivolumab	SMD
*n*	485	333	
Age at resection (mean [SD])	65.80 (8.57)	65.32 (8.27)	0.058
Sex (%)			
Male	381 (78.6)	279 (83.8)	0.134
Female	104 (21.4)	54 (16.2)	
Body mass index (median [IQR])	25.85 (4.13)	26.65 (3.98)	0.197
Charlson Comorbidity Index (median [IQR])	0.0 (0.0–1.0)	0.0 (0.0–1.0)	0.118
WHO performance status prior to nCRT (%)			
0	274 (56.5)	212 (63.7)	0.202
1	160 (33.0)	96 (28.8)	
2	16 (3.3)	5 (1.5)	
3	1 (0.2)	0 (0.0)	
4	0 (0.0)	1 (0.3)	
Missing	34 (7.0)	19 (5.7)	
Other malignancies^a^ (%)			
No	450 (92.8)	314 (94.3)	0.061
Yes	35 (7.2)	19 (5.7)	
Primary tumor location (%)			
Proximal or mid thoracic esophagus	38 (7.8)	24 (7.2)	0.100
Distal or unknown esophagus	391 (80.6)	280 (84.1)	
Junction or cardia	56 (11.5)	29 (8.7)	
Histology (%)			
Adenocarcinoma (intestinal type)	323 (66.6)	230 (69.1)	0.110
Adenocarcinoma (diffuse type)	47 (9.7)	35 (10.5)	
Adenocarcinoma (other/unknown type)	54 (11.1)	32 (9.6)	
Squamous cell carcinoma	59 (12.2)	33 (9.9)	
Other/Not otherwise specified	2 (0.4)	3 (0.9)	
Differentiation grade (%)			
1–2	263 (54.2)	173 (52.0)	0.103
3–4	189 (39.0)	128 (38.4)	
Unknown	33 (6.8)	32 (9.6)	
ypT (%)			
ypT0	15 (3.1)	22 (6.6)	0.249
ypT 1	104 (21.4)	74 (22.2)	
ypT 2	103 (21.2)	80 (24.0)	
ypT 3	249 (51.3)	151 (45.3)	
ypT 4	5 (1.0)	5 (1.5)	
Missing	9 (1.9)	1 (0.3)	
ypN (%)			
ypN0	233 (48.0)	148 (44.4)	0.138
ypN 1	154 (31.8)	108 (32.4)	
ypN 2	61 (12.6)	53 (15.9)	
ypN 3	35 (7.2)	24 (7.2)	
Missing	2 (0.4)	0 (0.0)	
Number of cycles carboplatin and paclitaxel (median [IQR])	5 (5–5)	5 (5–5)	0.127
Radiotherapy dose (median [IQR])	41.4 (41.4–41.4)	41.4 (41.4–41.4)	0.047
Days between nCRT and resection (median [IQR])	75.0 (62.0–92.3)	77.0 (64.0–91.3)	0.045
Type of surgery (%)			
Transhiatal esophagectomy	49 (10.1)	31 (9.3)	0.216
Ivor‐Lewis esophagectomy	320 (66.0)	229 (68.8)	
McKeown esophagectomy	90 (18.6)	55 (16.5)	
Other or unknown type of esophagectomy	15 (3.1)	17 (5.1)	
Gastrectomy	6 (1.2)	1 (0.3)	
Other	5 (1.0)	0 (0.0)	
Annual resection volume of hospital (%)			
<20	25 (5.2)	14 (4.2)	0.169
20–29	59 (12.2)	34 (10.2)	
30–39	58 (12.0)	44 (13.2)	
>40	338 (69.7)	241 (72.4)	
Missing	5 (1.0)	0 (0.0)	
Surgical radicality (%)			
R0	394 (81.2)	271 (81.4)	0.052
R1/2	68 (14.0)	43 (12.9)	
Missing	23 (4.7)	19 (5.7)	
Post‐operative complications (%)			
No grade 3/4 complication	377 (77.7)	276 (82.9)	0.188
Grade 3 complication	62 (12.8)	30 (9.0)	
Grade 4 complication	35 (7.2)	15 (4.5)	
Missing	11 (2.3)	12 (3.6)	

Abbreviations: IQR, Interquartile range; SMD, Standardized Mean Difference.

^a^ Other malignancies 12 months prior to or 12 months after diagnosis esophageal or gastroesophageal junction cancer.

**TABLE 2:** Patient characteristics of both treatment arms after propensity score matching.

	No Adjuvant nivolumab	Adjuvant nivolumab	SMD
*N*	308	308	
Age at resection (mean [SD])	65.70 (8.60)	65.62 (8.09)	0.010
Sex (%)			
Male	257 (83.4)	255 (82.8)	0.017
Female	51 (16.6)	53 (17.2)	
Body mass index (median [IQR])	26.26 [23.98, 28.66]	26.03 [24.29, 28.53]	0.031
Charlson Comorbidity Index (median [IQR])	0.0 (0.0–1.0)	0.0 (0.0–1.0)	0.005
WHO performance status prior to nCRT (%)			
0	210 (68.2)	207 (67.2)	0.021
1	93 (30.2)	96 (31.2)	
2	5 (1.6)	5 (1.6)	
Other malignancies^a^ (%)			
No	292 (94.8)	290 (94.2)	0.028
Yes	16 (5.2)	18 (5.8)	
Primary tumor location (%)			
Proximal or mid thoracic esophagus	17 (5.5)	20 (6.5)	0.066
Distal or unknown esophagus	258 (83.8)	260 (84.4)	
Junction or cardia	33 (10.7)	28 (9.1)	
Histology (%)			
Adenocarcinoma (intestinal type)	213 (69.2)	215 (69.8)	0.026
Adenocarcinoma (diffuse type)	33 (10.7)	31 (10.1)	
Adenocarcinoma (other/unknown type)	31 (10.1)	30 (9.7)	
Squamous cell carcinoma	29 (9.4)	30 (9.7)	
Other/Not otherwise specified	2 (0.6)	2 (0.6)	
Differentiation grade (%)			
1–2	167 (54.2)	165 (53.6)	0.024
3–4	117 (38.0)	117 (38.0)	
Unknown	24 (7.8)	26 (8.4)	
ypT (%)			
yp0	12 (3.9)	12 (3.9)	0.058
yp1	70 (22.7)	70 (22.7)	
yp2	68 (22.1)	76 (24.7)	
yp3	156 (50.6)	147 (47.7)	
yp4	2 (0.6)	3 (1.0)	
ypN (%)			
yp0	147 (47.7)	143 (46.4)	0.058
yp1	91 (29.5)	98 (31.8)	
yp2	47 (15.3)	47 (15.3)	
yp3	23 (7.5)	20 (6.5)	
Number of cycles carboplatin and paclitaxel (median [IQR])	5 (5–5)	5 (5–5)	0.010
Radiotherapy dose (median [IQR])	41.4 (41.4–41.4)	41.4 (41.4–41.4)	0.005
Days between nCRT and resection (median [IQR])	76.00 [64.75, 94.25]	77.00 [64.00, 92.00]	0.014
Type of surgery (%)			
Transhiatal esophagectomy	31 (10.1)	30 (9.7)	0.094
Ivor‐Lewis esophagectomy	215 (69.8)	211 (68.5)	
McKeown esophagectomy	48 (15.6)	52 (16.9)	
Other or unknown type of esophagectomy	13 (4.2)	15 (4.9)	
Annual resection volume of hospital (%)			
<20	17 (5.5)	13 (4.2)	0.069
20–29	31 (10.1)	32 (10.4)	
30–39	44 (14.3)	41 (13.3)	
>40	216 (70.1)	222 (72.1)	
Surgical radicallity (%)			
R0	275 (89.3)	267 (86.7)	0.080
R1/2	33 (10.7)	41 (13.3)	
Post‐operative complications (%)			
No grade 3/4 complication	272 (88.3)	266 (86.4)	0.072
Grade 3 complication	22 (7.1)	28 (9.1)	
Grade 4 complication	14 (4.5)	14 (4.5)	

Abbreviations: IQR, Interquartile range; SMD, Standardized Mean Difference.

^a^ Other malignancies 12 months prior to or 12 months after diagnosis esophageal or gastroesophageal junction cancer.

The included patient population was predominantly male (83%) and was on average 65 years old. The primary tumor was located in the distal (or unknown) part of the esophagus in the majority of patients (84%) and most often was an adenocarcinoma (90%) (Table 2). With regard to the nCRT and surgery, 86% of the patients completed all 5 cycles of carboplatin and paclitaxel and 41.4 Gy radiotherapy, while 71% of the patients underwent surgery in a hospital with ≥40 annual esophagectomies, 87% had an R0 resection, and 87% had no grade 3 or 4 post‐operative complication.

**FIGURE 1:**
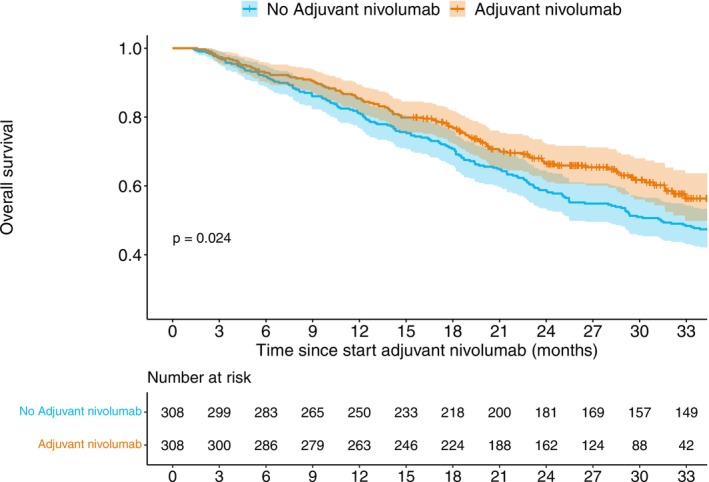
Overall survival analyses on adjuvant nivolumab versus no adjuvant nivolumab groups.

Median follow‐up time was 24.4 (Interquartile range [IQR]: 17.3–30.9) months and 31.4 (IQR: 15.3–43.4) months for patients treated with and without adjuvant nivolumab, respectively. The median survival times for the group treated with and without adjuvant nivolumab were 36.2 months (95% CI: 33‐NA) and 31.4 months (95% CI: 25.5–37.4), respectively. The 2‐year OS was 66.8% (95% CI: 61.6%–72.4%) for the nivolumab group and 58.8% (95% CI: 53.5%–64.5%) for the group without nivolumab (log‐rank *p* = 0.024) (Figure 1 and Table 3). The univariable hazard ratio for OS with nivolumab versus without nivolumab treatment was 0.75, 95% CI: 0.60–0.97, demonstrating favorable survival for patients treated with adjuvant nivolumab.

**TABLE 3:** Overall survival at 6, 12, and 18 months.

Time since start adjuvant nivolumab	No adjuvant nivolumab		Adjuvant nivolumab	
	Number at risk	OS (95% CI)	Number at risk	OS (95% CI)
6 months	283	91.9 (88.9–95.0)	286	92.9 (90.0–95.8)
12 months	250	81.2 (76.9–85.7)	263	85.4 (81.5–89.4)
18 months	218	70.8 (65.8–76.0)	224	77.2 (72.6–82.0)
24 months	181	58.8 (53.5–64.5)	162	66.8 (61.6–72.4)
30 months	157	51.0 (45.7–56.9)	88	61.7 (56.1–70.0)

**FIGURE 2:**
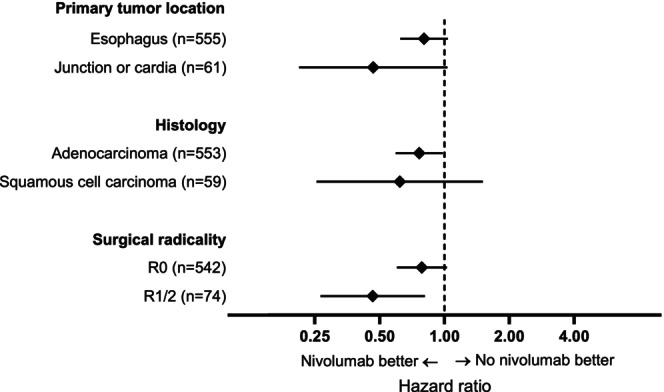
Subgroup analyses on overall survival of adjuvant nivolumab versus no adjuvant nivolumab.

Subgroup analyses showed a significant OS difference for patients with adenocarcinoma treated with nivolumab, with a hazard ratio of 0.76 (95% CI: 0.59–0.98, *p =* 0.04), and for patients who had irradical (R1/R2) surgical resection and were treated with nivolumab, with a hazard ratio of 0.46 (95% CI: 0.27–0.81, *p* = 0.007). No significant difference was observed in any of the other subgroups (Figure 2). All interaction effects between adjuvant nivolumab and histology, surgical radicality, and primary tumor location were not significant (Table S1).

Sensitivity analysis of the observed treatment effect revealed that the E‐value was 1.74.

## 4 DISCUSSION

In this nationwide real‐world propensity score matched study with a median follow‐up of 24 months of the nivolumab group, we found a significantly higher overall survival for patients with non‐metastatic esophageal or gastroesophageal cancer who received adjuvant nivolumab compared to patients who did not receive adjuvant nivolumab after an incomplete pathologic response to nCRT.

The CheckMate‐577 trial reported 2‐year OS rates of 68% and 66% for the nivolumab and placebo arm. Notably, the OS difference widened after year 2, stabilizing at a consistent difference of approximately 5% to 7% from year 3 onward.^8^ In our study, 2‐year OS rates were 67% for patients treated with adjuvant nivolumab and 59% for those who were not, closely matching the absolute OS levels reported in the CheckMate‐577 trial at year 2. Although outcomes with new therapies are often less favorable in routine clinical practice than in clinical trials, the OS results in our current study appear broadly comparable to those reported in the CheckMate‐577 trial.

A possible explanation for this may lie in the rigorous adherence to the CROSS protocol for nCRT in our study: 86% of patients completed all five cycles of carboplatin and paclitaxel with 41.4 Gy radiotherapy. In contrast, nCRT in the CheckMate 577 trial was more heterogeneous, with only 71% receiving carboplatin and paclitaxel, an unreported number of chemotherapy cycles, and 63% receiving radiotherapy doses between 41.4 and 50.4 Gy. Additionally, 71% of patients in our study underwent surgery in hospitals performing ≥40 esophagectomies per year, whereas hospital resection volumes were not reported in CheckMate 577. These factors may reflect a relatively high survival baseline in Dutch routine clinical practice.

The CheckMate‐577 trial highlighted a greater DFS and OS benefit in the squamous cell carcinomas compared to the adenocarcinomas. Conversely, patients with GEJ cancer exhibited a lower DFS and no OS benefit of adjuvant treatment with nivolumab compared to patients with the primary tumor located in the esophagus. This is in contrast to our subgroup analyses in which we found a significantly higher survival for patients with adenocarcinoma but not for patients with a squamous cell carcinoma and no indication that patients with a GEJ would have a lower benefit compared to patients with the primary tumor located in the esophagus. It is important to note that some groups in our study were relatively small, especially the squamous cell carcinoma group, and when combined with limited follow‐up, this resulted in constrained statistical power. Consequently, the findings from the subgroup analyses should be interpreted with considerable caution.

In general, we do not know whether treating earlier means treating better. Adjuvant treatment has the goal of curing patients with undetectable micro metastases. Monotherapy checkpoint inhibition in first line for metastatic gastroesophageal cancer failed to show survival gain.^15^ In combination with chemotherapy, checkpoint inhibition has shown improved survival outcomes in patients with PD‐L1 positive tumors.^16^, ^17^ At this time, it is unknown whether adjuvant monotherapy with nivolumab to treat undetected micro metastases is beneficial in terms of OS gain over starting treatment at the time of diagnosis of metachronous metastatic disease. This study cannot answer that question, as patients in our study who were not treated with adjuvant nivolumab most likely only received chemotherapy upon disease recurrence, as immune checkpoint inhibition was not reimbursed yet in the metastatic setting.

With the recent publications of the results of the ESOPEC, Matterhorn, and CheckMate‐577 trials, the treatment landscape of gastroesophageal cancer is rapidly evolving.^8^, ^18^, ^19^ As a direct comparison between adjuvant nivolumab after nCRT and resection versus a perioperative FLOT regimen is still lacking, the most effective perioperative strategy for patients with gastroesophageal cancer remains uncertain. However, given that a substantial proportion of these patients in daily clinical practice are elderly and may have comorbidities, limiting their ability to tolerate intensive three‐drug chemotherapy regimens (with or without durvalumab), it is clinically relevant to note that the less intensive approach of CROSS chemoradiotherapy followed by surgery—and adjuvant nivolumab in cases of incomplete pathological response—has shown an OS benefit in routine clinical practice.

Several limitations of our study should be discussed. First, adjuvant treatment nivolumab was only available since 2022 in the Netherlands, which resulted in short follow‐up compared to patients who received nCRT and resection. Potential long‐term outcomes of patients treated with nivolumab in the adjuvant setting in our real‐world cohort could not yet be investigated. Providing insights into the possible overall survival benefits of adjuvant nivolumab treatment can help clinicians and patients make more informed decisions and may contribute to the ongoing evaluation and optimization of treatment strategies for esophageal cancer. Therefore, publishing our data, despite its limitations, is highly relevant and necessary to advance the understanding and management of esophageal cancer.

Second, despite the construction of two comparable treatment groups based on factors which are known to be related to survival outcomes and treatment allocation, patients were obtained from real‐world observational data. Thus, despite our efforts, there might be some unmeasured residual confounding, and results should be interpreted with caution. However, sensitivity analyses of the robustness of the treatment effects revealed that a potential unobserved confounder would need to have a HR of 1.74 or larger to negate the treatment effect. Given that we have accounted for most of the known clinical confounders, it is unlikely that there is an unobserved confounder with a HR of 1.74 or higher that would thereby entirely negate the treatment effect. Third, as the collection of data on DFS and subsequent therapies requires additional manual data registration by data managers of the NCR, we currently lack comprehensive data on DFS and subsequent therapies for the patients in our study. The patients treated without adjuvant nivolumab largely had their disease relapse in an era in which ICIs in the metastatic setting were not yet reimbursed, in contrast to those treated with adjuvant nivolumab. As the addition of ICI to chemotherapy increased survival in the biomarker‐positive subgroups in the registration trials,^16^, ^17^ this may have positively impacted the post‐recurrence survival of the adjuvant nivolumab group, and thus lead to imbalances in the two groups. Finally, data on CPS in the resection specimen were not available, precluding subgroup analyses by PD‐L1 expression, such as comparisons of OS between patients with CPS ≥1 and those with CPS <1, which could have provided insight into the differential benefit from adjuvant nivolumab.

A strength of our study is the thorough identification and inclusion of most of the pertinent measurable factors related to survival and treatment allocation, achieved through the utilization of expert knowledge from physicians and healthcare professionals. This approach allowed us to successfully balance treatment groups, thereby minimizing the risk of confounding as much as possible. Another strength of our study lies in the iterative comparisons between matching methods to determine the matching method that yielded the highest number of matched patients and achieved optimal covariate balance.

In conclusion, our study revealed a significantly higher OS in patients with esophageal or GEJ cancer treated with adjuvant nivolumab after nCRT and resection compared to patients without adjuvant treatment in a real‐world matched comparison after a median of 24 months of follow‐up for the nivolumab group. Given the current limitations in follow‐up duration, the relatively low number of events, the possible residual confounding, and the possible differences in post‐recurrence treatment, these findings should be approached with caution and warrant reevaluation in the coming years as additional data accrue.

## AUTHOR CONTRIBUTIONS

**Rob H. A. Verhoeven:** Conceptualization; methodology; data curation; supervision; formal analysis; writing – original draft; writing – review and editing; investigation; software. **Steven C. Kuijper:** Methodology; data curation; formal analysis; writing – original draft; writing – review and editing; investigation; conceptualization; software. **Marije Slingerland:** Writing – review and editing. **Bas Wijnhoven:** Writing – review and editing. **Mark I. van Berge Henegouwen:** Writing – review and editing. **Peter S. N. van Rossum:** Writing – review and editing. **Sarah Derks:** Writing – review and editing. **Bianca Mostert:** Writing – review and editing. **Nadia Haj Mohammad:** Writing – review and editing. **Hanneke W. M. van Laarhoven:** Conceptualization; supervision; methodology; writing – review and editing; validation; investigation.

## CONFLICT OF INTEREST STATEMENT

Rob Verhoeven: Received research grant from Bristol Myers Squibb and served as consultant for Daiichi Sankyo Inc., all paid to institution. Marije Slingerland: reports advisory/speaker role from BMS, Lilly, and AstraZeneca. Bas Wijnhoven: reports research grant and consulting fee form BMS. Mark van Berge Henegouwen: proctor for Intuitive, consultancy for Medtronic, Johnson and Johnson, BBraun, Stryker, all fees paid to the institution. Bianca Mostert: Research funding from Pfizer and BMS, consulting/advisory role for Lilly, Servier, BMS, Astra Zeneca, and Amgen. Nadia Haj Mohammad: Consultancy/advisory role from: Servier, AstraZeneca, Bristol Myers Squibb/Pfizer, Merck/Pfizer, and Lilly. Hanneke van Laarhoven: Research funding and/or medication/material supply from: AMGEN, Auristone, BMS, Incyte, Merck, MyeloidTx, ORCA, Servier. Consultant/advisory role: AMGEN, Amphera, Astellas, AstraZeneca, Beigene, BMS, Boehringer, Daiichy, MSD, MyeloidTx. Speaker role: AstraZeneca, BMS, Congress Care, Daiichy, Medtalks, Uitgeverij JAAP, Travel Congress Management. Sarah Derks: Research funding from INCYTE, advisory role/speaker role from BMS, Servier, Benecke. Steven Kuijper and Peter van Rossum declare no competing interests.

## ETHICS STATEMENT

According to the Central Committee on Research involving Human Subjects, this type of study does not require approval from an ethics committee in the Netherlands. Our study was approved by the Privacy Review Board of the NCR and the scientific committee of the Dutch Upper GI Cancer Group.

## Supporting information


**TABLE S1.** Results of testing of interaction effects on overall survival between adjuvant nivolumab treatment with histology, surgical radicality and primary tumor location.

## Data Availability

The data underlying this article is available at the Netherlands Comprehensive Cancer Organisation (IKNL) according to regular request and approval procedures. More information is available at: https://iknl.nl/international/data-request. Further information is available from the corresponding author upon request.
