# Supplementary material for: Adjuvant nivolumab after chemoradiotherapy and resection for patients with esophageal cancer: A real‐world matched comparison of overall survival
Source: Int J Cancer. 2025 Sep 23;158(5):1292–301. doi: 10.1002/ijc.70168 (PMC12765972; doi:10.1002/ijc.70168)
Supplement: Supplementary file 1 — TABLE S1. Results of testing of interaction effects on overall survival between adjuvant nivolumab treatment with histology, surgical radicality and primary tumor location. [file IJC-158-1292-s001.pdf]

Supplementary material

Adjuvant nivolumab after chemoradiotherapy and resection for patients with esophageal cancer: a real-world matched comparison of overall survival

Rob H.A. Verhoeven PhD, Steven C. Kuijper MSc, Marije Slingerland PhD MD, Bas Wijnhoven PhD MD, Mark I. van Berge Henegouwen PhD MD, Peter S.N. van Rossum PhD MD, Sarah Derks PhD MD, Bianca Mostert PhD MD, Nadia Haj Mohammad PhD MD, Hanneke W.M. van Laarhoven PhD MD

Content of supplementary material

Supplementary table 1

Supplementary table 1, Results of testing of interaction effects on overall survival between adjuvant nivolumab treatment with histology, surgical radicality and primary tumor location.

**Model**	**Parameter**		**HR (95%CI)**	**P value**
**Surgical radicality**				
	Adjuvant nivolumab	No (REF)	-	-
		Yes	0.78 (0.62-1.02)	0.072
	Surgical radicality	0 (REF)	-	-
		1	2.58 (1.71-3.82)	<0.001
	Adjuvant nivolumab * Surgical radicality		0.65 (0.35-1.21)	0.173
**Primary tumor location**				
	Adjuvant nivolumab	No (REF)	-	-
		Yes	0.79 (0.54-1.02)	0.071
	Primary tumor location	Junction or cardia (REF)	-	-
		Esophagus	1.13 (0.71-1.80)	0.609
	Adjuvant nivolumab * Primary tumor location		0.63 (0.28-1.45)	0.280
**Histology**				
	Adjuvant nivolumab	No (REF)	-	-
		Yes	0.76 (0.60-0.97)	0.029
	Histology	Adenocarcinoma (REF)	-	-
		Squamous cell carcinoma	0.77 (0.44-1.33)	0.355
	Adjuvant nivolumab * Histology		0.90 (0.37-2.22)	0.825

HR = Hazard Ratio
